# Developing Synergistic Drug Combinations To Restore Antibiotic Sensitivity in Drug-Resistant Mycobacterium tuberculosis

**DOI:** 10.1128/AAC.02554-20

**Published:** 2021-04-19

**Authors:** Charles Omollo, Vinayak Singh, Elizabeth Kigondu, Antonina Wasuna, Pooja Agarwal, Atica Moosa, Thomas R. Ioerger, Valerie Mizrahi, Kelly Chibale, Digby F. Warner

**Affiliations:** a Department of Chemistry, University of Cape Town, Rondebosch, South Africa; b South African Medical Research Council Drug Discovery and Development Research Unit, University of Cape Town, Rondebosch, South Africa; c SAMRC/NHLS/UCT Molecular Mycobacteriology Research Unit, DSI/NRF Centre of Excellence for Biomedical Tuberculosis Research, Department of Pathology, University of Cape Town, Rondebosch, South Africa; d Institute of Infectious Disease and Molecular Medicine, University of Cape Town, Rondebosch, South Africa; e Texas A&M University, Department of Computer Science, College Station, Texas, USA; f Wellcome Centre for Infectious Diseases Research in Africa, University of Cape Town, Rondebosch, South Africa

**Keywords:** Rv1258c, chlorpromazine, efflux, fusidic acid, potentiation, spectinomycin

## Abstract

Tuberculosis (TB) is a leading global cause of mortality owing to an infectious agent, accounting for almost one-third of antimicrobial resistance (AMR) deaths annually. We aimed to identify synergistic anti-TB drug combinations with the capacity to restore therapeutic efficacy against drug-resistant mutants of the causative agent, Mycobacterium tuberculosis. We investigated combinations containing the known translational inhibitors, spectinomycin (SPT) and fusidic acid (FA), or the phenothiazine, chlorpromazine (CPZ), which disrupts mycobacterial energy metabolism. Potentiation of whole-cell drug efficacy was observed in SPT-CPZ combinations. This effect was lost against an M. tuberculosis mutant lacking the major facilitator superfamily (MFS) efflux pump, Rv1258c. Notably, the SPT-CPZ combination partially restored SPT efficacy against an SPT-resistant mutant carrying a g1379t point mutation in *rrs*, encoding the mycobacterial 16S rRNA. Combinations of SPT with FA, which targets the mycobacterial elongation factor G, exhibited potentiating activity against wild-type M. tuberculosis. Moreover, this combination produced a modest potentiating effect against both FA-monoresistant and SPT-monoresistant mutants. Finally, combining SPT with the frontline anti-TB agents, rifampicin (RIF) and isoniazid, resulted in enhanced activity *in vitro* and *ex vivo* against both drug-susceptible M. tuberculosis and a RIF-monoresistant *rpoB* S531L mutant. These results support the utility of novel potentiating drug combinations in restoring antibiotic susceptibility of M. tuberculosis strains carrying genetic resistance to any one of the partner compounds.

## Abstract (precis)

Tuberculosis (TB) is a leading global cause of mortality owing to an infectious agent, accounting for almost one-third of antimicrobial resistance (AMR) deaths annually. We aimed to identify synergistic anti-TB drug combinations with the capacity to restore therapeutic efficacy against drug-resistant mutants of the causative agent, Mycobacterium tuberculosis.

## INTRODUCTION

The increasing prevalence of multidrug-resistant tuberculosis (MDR-TB)—defined as resistance to the frontline anti-TB agents isoniazid (INH) and rifampicin (RIF)—necessitates the urgent development and implementation of new antimycobacterial drugs and therapeutic strategies (1, 2). A number of anti-TB compounds are currently in the drug discovery pipeline, with several others in advanced preclinical development (3). However, with the exception of bedaquiline (BDQ) and delamanid, no TB-specific drugs have been introduced into clinical use within the past 40 years (4). Therefore, new options need to be explored to address the problem of drug resistance.

Novel combination regimens comprising standard anti-TB agents and repurposed drugs represent a logical approach, especially where the new partner drug has already been approved for other clinical indications (5–7). Recent advances in understanding the physicochemical properties that determine drug distributions within complex tissue and cellular (micro)environments (8, 9), together with improved methods for rapid selection of multiple potentially synergistic drug partners *in vitro* and *in vivo* (8, 10–12), suggest the potential for rational development of novel combination approaches. This is important since it might address the long-held belief that developing combinations should be avoided owing to the complexities inherent in ensuring simultaneous and sustained delivery of the optimal partner compounds to the same target site (11). The impact of preexisting drug resistance on the utility of new drug combinations presents an additional challenge and is of particular concern when these combinations comprise current frontline anti-TB agents. To minimize the risks of exposing an individual to effective monotherapy, the likely preexistence of resistance to individual drugs must be recognized and informed combination approaches for drug therapies designed. These should incorporate multiple attributes beyond simply selecting individual molecules based on their biological activities as single agents (13).

In this study, we employed spectinomycin (SPT) as an anchor compound in combination with other experimental antibiotics and existing frontline anti-TB agents. SPT is an aminocyclitol antibiotic that inhibits protein synthesis by disrupting mRNA interactions with the 30S ribosome (14). Unlike other aminocyclitol antibiotics (including gentamicin and kanamycin [KAN]), SPT is not ototoxic (15) and has been used extensively in treating Neisseria gonorrhoeae infections in patients who cannot tolerate first-line treatments (16). From the perspective of new regimen design, SPT has been shown in combination screens against M. tuberculosis to synergize with several different classes of antimycobacterial compounds, both *in vitro* and in a macrophage model (10). Unfortunately, a key liability undermining its utility as a single agent is that SPT is subject to active efflux by M. tuberculosis—an observation that motivated an elegant medicinal chemistry solution in the development of the spectinamides (SPD) as derivative “efflux-resistant” anti-TB antibiotics (17–19). Spectinamides are also known to synergize with a variety of antibiotic classes (11), with lead spectinamide molecules, such as 1599, shown to be active against MDR M. tuberculosis strains and to synergize with existing and experimental anti-TB drugs *in vivo* (11, 20). However, developing suitable spectinamide formulations for therapeutic delivery remains an obstacle to the advancement of these compounds as novel anti-TB agents (21, 22).

We investigated the utility of potentiating combinations, anchored by SPT, to circumvent drug resistance and potentially restore (partial) susceptibility where genetically drug-resistant mutants preexist for one of the partner compounds. We applied combination screens utilizing (i) chlorpromazine (CPZ), a phenothiazine whose complex and unresolved mechanism of action involving disruption of the mycobacterial electron transport chain (23) has been implicated in efflux pump inhibition (24), and (ii) fusidic acid (FA), a translational inhibitor with demonstrated (albeit moderate) activity *in vitro* (25, 26). FA was selected owing to its potential for repositioning as anti-TB agent and because it possesses a unique mechanism of action, specifically, inhibition of bacterial protein synthesis by binding to elongation factor G (EF-G) (27). The antimicrobial-potentiating effect of FA with other antibiotics, including the frontline anti-TB drug ethambutol (EMB), as well as its lack of cross-resistance to other antimicrobial classes, provided additional motivation for our choice of FA (12, 28). By testing these combinations against both drug-susceptible M. tuberculosis H37Rv and selected drug-resistant mutants, we explored new potentiating combinations and demonstrated the utility of developing potent combinations against bacilli carrying preexisting genetic resistance to either of the partner drugs. In addition, this work revealed that the addition of SPT as third agent to the existing first-line anti-TB drug combination of RIF and INH restores activity *in vitro* against defined pre-MDR mutants of M. tuberculosis.

## RESULTS

### CPZ potentiates SPT activity by inhibiting Rv1258c-mediated efflux.

**FIG 1:**
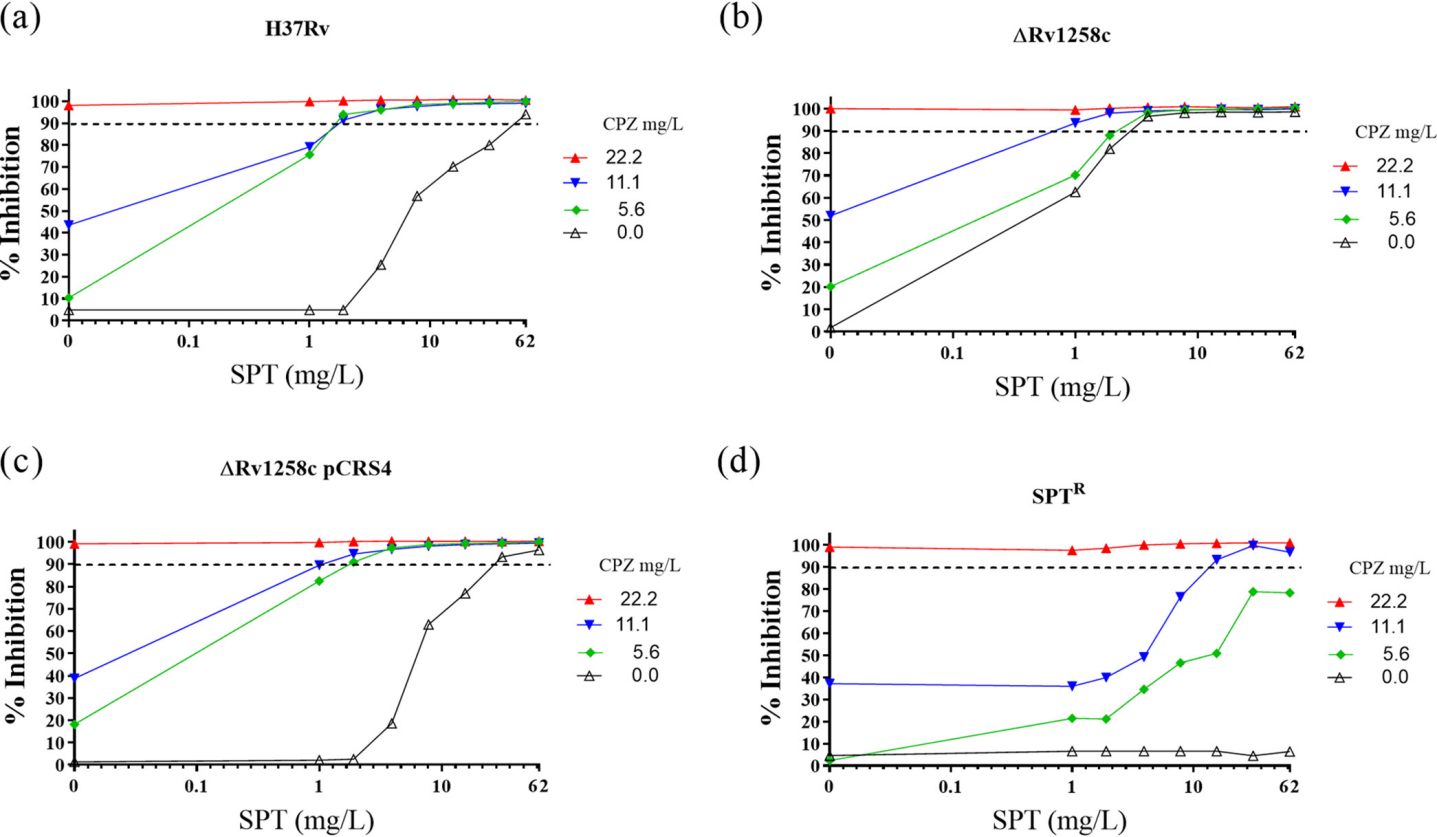
Inhibition of Rv1258c-mediated efflux of SPT by addition of CPZ. Combinations of CPZ and SPT were applied in checkerboard assays against wild-type M. tuberculosis H37Rv (a), the ΔRv1258c mutant (b), the ΔRv1258c pCRS4 complemented mutant (c), and the SPT^r^ strain (d). Bacterial growth inhibition was assessed in two independent experiments by fluorescence-based resazurin assay. The dashed horizontal line indicates 90% inhibition, and data are the means and standard deviations of two independent biological replicates.

The combination of SPT and CPZ was previously reported as synergistic against Mycobacterium smegmatis (29). When tested against wild-type M. tuberculosis H37Rv (Fig. 1a), the same combination yielded a sum of fractional inhibitory concentration (ΣFIC) value of 0.09 (Table 1), confirming strong synergy (30). We investigated whether this effect resulted from CPZ-mediated disruption of the activity of the major facilitator superfamily (MFS) efflux pump, Rv1258c, which has been implicated in innate resistance to SPT (18). We performed checkerboard assays using the efflux-defective ΔRv1258c (“tap”) mutant, which had been used in the development of the spectinamides (SPD) (18), and its complemented derivative, ΔRv1258c pCRS4. Both the ΔRv1258c mutant and the complemented mutant exhibited the same MIC_90_ of 22 mg/liter for CPZ (Fig. 1 and Table 1), whereas the ΔRv1258c mutant was hypersusceptible to SPT, displaying an approximately 6-fold lower MIC_90_ of 3.9 mg/liter (Fig. 1b and Table 1; see also Table S1 in the supplemental material) as observed previously (31). Notably, the synergy detected on exposing wild-type M. tuberculosis to a combination of CPZ and SPT (Fig. 1a) was eliminated in the ΔRv1258c mutant (Fig. 1b)—which yielded a ΣFIC value of 0.75 (Table 1)—but was restored in the complemented ΔRv1258c pCRS4 strain (Fig. 1c), with a ΣFIC of 0.12 (Table 1). Previous studies have reported no significant alteration in Rv1258c transcription in response to CPZ treatment (32). Therefore, our observations suggested that CPZ treatment abrogated efflux-mediated intrinsic resistance to SPT in wild-type M. tuberculosis in a manner dependent on Rv1258c, perhaps as a result of CPZ-mediated inhibition of energy metabolism (23).

**TABLE 1:** Investigation of potential synergies between SPT and CPZ against different M. tuberculosis strains through the calculation of the FIC and sum of the FIC

M. tuberculosis strain or mutant	Drug combination	MIC (mg/liter)^*a*^		ΣFIC^*b*^	Corresponding Fig. 1 panel^*c*^
		Alone	In combination		
H37Rv	SPT	62	1.9	0.09	a
	CPZ	22.2	1.4		
ΔRv1258c	SPT	3.9	1.0	0.76	b
	CPZ	22.2	11.1		
ΔRv1258c pCRS4	SPT	31.0	1.9	0.12	c
	CPZ	22.2	1.4		
SPT^r^	SPT	>248	15.5	0.56	d
	CPZ	22.2	11.1		

a The MIC was defined as the lowest drug concentration that inhibited growth by at least 90%.

b The FIC of each drug was calculated as follows: (MIC of drug in combination)/(MIC of individual drug). The ΣFIC is the sum of the FICs of the two drugs where a ΣFIC of ≤0.5 is synergistic, ≥4.0 is antagonistic, and any value in the range 0.5 <* x *< 4.0 is considered additive or no interaction (64).

c The respective figure panels show data of drug concentrations from which corresponding FIC values are calculated and the resulting ΣFIC computed.

### CPZ synergizes with compounds other than SPT, but SPT potentiation arises solely from CPZ-mediated inhibition of Rv1258c.

To determine whether the synergy observed was specific for SPT or might apply to other antimycobacterial agents, CPZ was applied as the anchor compound in combination assays with a panel of anti-TB antibiotics of different classes and mechanisms of action (see Table S2 in the supplemental material). Of the eight compounds tested with CPZ, four exhibited clear synergy (ΣFIC ≤ 0.5) as follows: the frontline agents, RIF and INH, which returned ΣFICs of 0.37 and 0.5, respectively, and bedaquiline (BDQ) and nalidixic acid, both of which gave ΣFICs of ≤0.25. In contrast, no potentiation was observed with KAN or the fluoroquinolones, ciprofloxacin (CIP) and levofloxacin (LEV), all of which yielded ΣFICs of >0.5.

In a complementary approach, we also investigated if the potentiating effect observed with the SPT-CPZ combination was unique to CPZ. To this end, we assayed SPT in combination with an expanded panel of antimycobacterial agents (see Fig. S2 and Table S3 in the supplemental material). SPT was found to synergize with only 2 of the 11 compounds tested as follows: erythromycin (ERY), a macrolide targeting the ribosome, and verapamil (VER), a cationic amphiphile that was originally considered an M. tuberculosis efflux pump inhibitor but has been shown recently to disrupt membrane function (33). RIF, the mycobacterial RNA polymerase inhibitor, was just beyond the threshold determining synergistic activity.

To ascertain if inhibition of Rv1258c-mediated efflux resulted in the observed compound synergies, the ΔRv1258c mutant was tested for hypersensitivity to a corresponding panel of anti-TB agents (Fig. S3 and Table S1 in the supplemental material). The spectinamide 1599 had the same MIC_90_ for both wild-type M. tuberculosis H37Rv and the Rv1258c mutant, reflecting its successful modification to avoid Rv1258c-mediated efflux (18). Of the other 11 compounds tested, only SPT was associated with hypersensitivity in the Rv1258c-knockout mutant, returning an MIC_90_ value of 0.39 mg/liter compared to the MIC_90_ value of 62 to 125 mg/liter against the wild-type strain. In combination, these results strongly support the inference that the synergy detected with the CPZ-SPT combination arises from CPZ-mediated inhibition of Rv1258c.

### The CPZ-SPT combination partially restores SPT sensitivity in an SPT-resistant mutant.

A spontaneous SPT-resistant mutant (SPT^r^) carrying a g1379t point mutation in the mycobacterial 16S rRNA, *rrs*, was associated with a >64-fold increase in the SPT MIC_90_ (see Table S1). In contrast, the activity of CPZ remained at the wild-type concentration for this strain, consistent with a mechanism of action of CPZ that was independent of *rrs* inhibition (34). While the SPT^r^ mutant was resistant to SPT at concentrations of >248 mg/liter in the absence of CPZ, combinations utilizing CPZ at sub-MICs ([CPZ] ≤ 11.1 mg/liter) restored SPT sensitivity, at least partially (Fig. 1d). These results suggested the capacity for synergistic combinations to (partially) restore drug activity against mutant strains genetically resistant to either of the partner compounds.

### Assessing synergy with SPT and fusidic acid, two antibiotics acting on the mycobacterial translational machinery.

The systematic application of drug combinations can reveal synergistic interactions. One form of synergy occurs when drug(s) that perturb normal cell physiology trigger (compensatory) cellular responses that can, in turn, affect (potentiate) the activities of other drugs (35). Nichols et al. demonstrated that the synergistic interaction between sulfamethoxazole and trimethoprim was a result of the two drugs targeting tetrahydrofolate biosynthesis (36). With this in mind, a combination comprising SPT and FA—translation inhibitors, which act at discrete steps of the elongation process—was tested against wild-type M. tuberculosis and two resistant strains, FA^r^ and SPT^r^ mutants. Isolation of spontaneous FA^r^ mutants *in vitro* yielded a single strain on 25× MIC FA at a frequency of ∼10^−8^. Whole-genome sequencing identified a c1384t (H462Y) substitution in *fusA1* (*Rv0684*), encoding the essential mycobacterial elongation factor G (EF-G) (37). The histidine residue is highly conserved across multiple bacterial species; therefore, using the Thermus thermophilus structure as template (38), it can be inferred that M. tuberculosis H462 corresponds to T. thermophilus H458 (39), mutations of which are likely to alter the FA-binding pocket (40). In MIC assays, the H462Y mutant consistently yielded an MIC_90_ of ≥25 mg/liter, confirming heritable FA^r^ (Table 2).

**TABLE 2:** Investigation of potential synergies between SPT and FA against different M. tuberculosis strains through the calculation of the FIC and sum of the FIC

M. tuberculosis strain	Drug combination	MIC (mg/liter)^*a*^		ΣFIC^*b*^	Corresponding Fig. 2 panel^*c*^
		Alone	In combination		
H37Rv	SPT	62	16.3	0.5	a
	FA	0.63	0.16		
SPT^r^	SPT	62	62	1.5	b
	FA	0.63	0.32		
FA^r^	SPT	62	31.5	1.5	c
	FA	25	25		

a The MIC was defined as the lowest drug concentration that inhibited growth by at least 90%.

b The FIC of each drug was calculated as follows: (MIC of drug in combination)/(MIC of individual drug). The ΣFIC is the sum of the FICs of the two drugs where a ΣFIC of ≤0.5 is synergistic, ≥4.0 is antagonistic, and any value in the range 0.5 <* x *< 4.0 is considered additive or no interaction (64).

c The respective figure panels show data of drug concentrations from which corresponding FIC values are calculated and the resulting ΣFIC computed.

**FIG 2:**
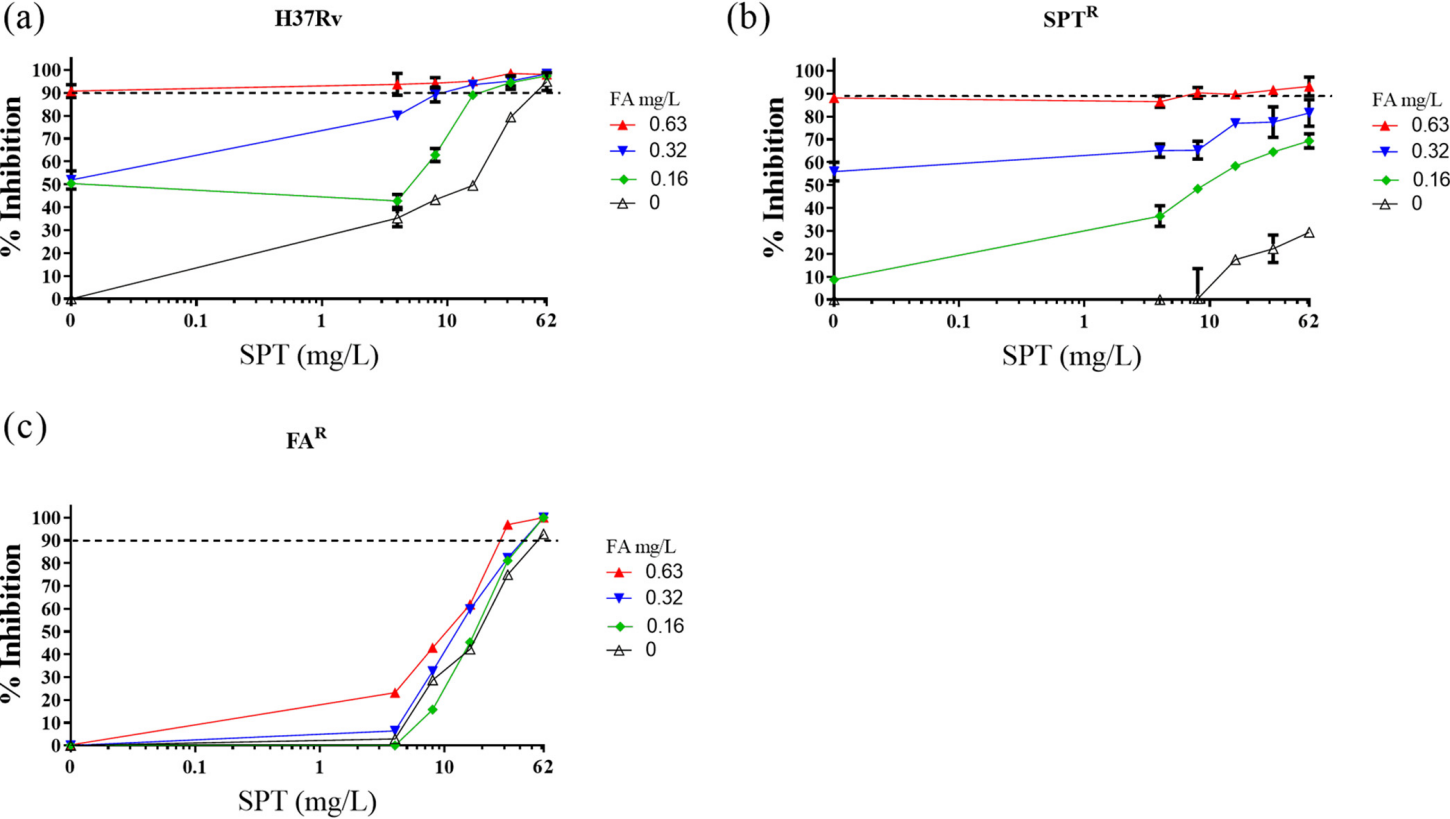
*In vitro* interaction between SPT and FA. Combinations of SPT and FA were applied in checkerboard assays against wild-type M. tuberculosis H37Rv (a), the SPT^r^ mutant (b), and the FA^r^ mutant (c). Bacterial viability was assessed by fluorescence-based resazurin assay. Dashed horizontal lines indicate 90% inhibition, and data are the means and standard deviations of two independent biological replicates.

With these strains in hand, we evaluated the interaction between SPT and FA and, furthermore, assessed whether this combination—which is synergistic against the parental, drug-susceptible M. tuberculosis H37Rv—might counter preexisting genetic resistance to either compound. The combination of SPT and FA exhibited a ΣFIC value of 0.50 (Table 2) against wild-type H37Rv; upon addition of FA at sub-MIC ([FA] = 0.16 mg/liter), the MIC_90_ of SPT exhibited an ∼4-fold decrease from 62 mg/liter to 16.3 mg/liter (Fig. 2a), *P* < 0.001. FA at subinhibitory concentration ([FA] = 0.32 and [FA] = 0.16 mg/liter) enhanced the activity of SPT ([SPT] = 62 mg/liter) against an SPT^r^ mutant. The inhibitory effect was significantly different from the results observed with similar concentrations of SPT in the absence of FA ([FA] = 0 mg/liter), *P* < 0.001 (Fig. 2b). Although the calculated sum FIC did not satisfy the criterion for “synergistic” (ΣFIC ≤ 0.5) (Table 2), the effect was marked and reproducible in two independent biological replicates (Fig. 2b). Notably, the same combination did not return enhanced activity against the FA^r^ mutant (Fig. 2c), strongly suggesting that FA was the major contributor to the SPT-FA combination. A summary of the inhibitory effects of the CPZ-SPT and FA-SPT combinations is presented in Table 3.

**TABLE 3:**
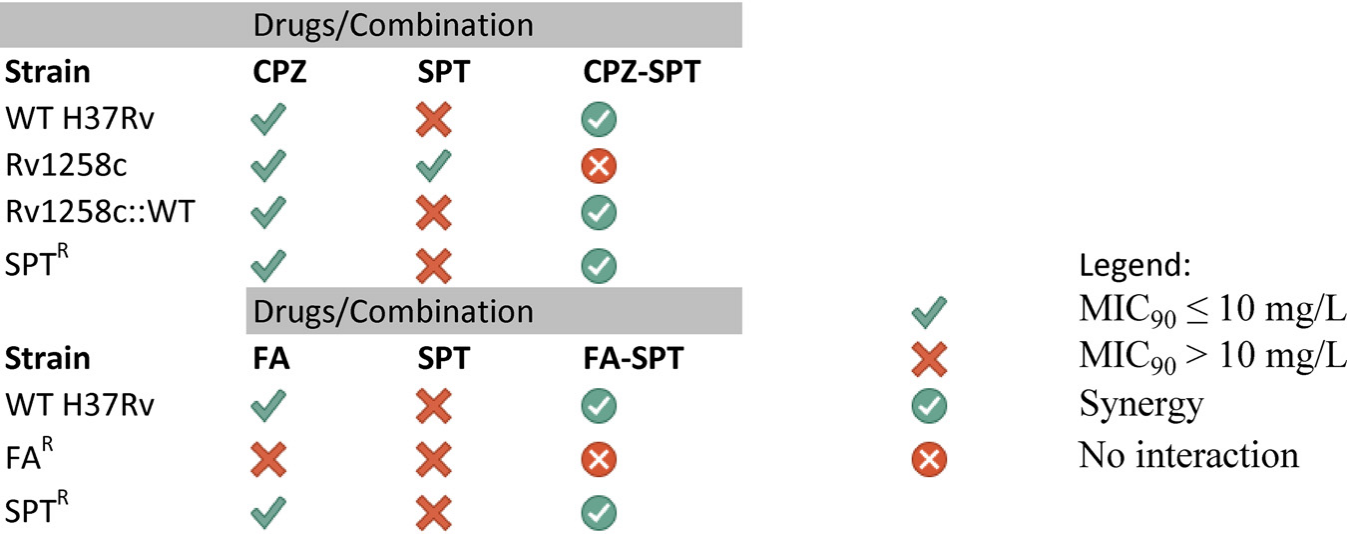
Summary of *in vitro* drug activities against M. tuberculosis H37Rv strains

### Confirmation that fluorescence intensities correlate with cell density readings.

The centrality of the resazurin microtiter assay (REMA) in determining the synergies reported in this study demanded orthogonal evidence supporting the claimed results. To this end, a 96-well-based antimycobacterial assay was performed with a selected panel of drugs having different mechanisms of action (see Fig. S4 in the supplemental material). The experiments were conducted using the H37Rv::GFP reporter mutant in which expression of the fluorophore is constitutive (41). After 8 days of incubation in the presence of 2-fold dilutions of the antimicrobial agents, green fluorescent protein (GFP) and resazurin fluorescence intensities were determined, and the corresponding optical density readouts recorded in parallel. The values of the respective fluorescence intensities and that of optical density at 600 nm (OD_600_) were converted to percent inhibition. Strong agreement was generally discerned when comparing GFP and OD_600_ methods with the standard resazurin readout (Fig. S4), supporting the use of the resazurin assay for both MIC and FIC determinations.

### A three-drug combination comprising SPT, RIF, and INH enhances *in vitro* activity against M. tuberculosis.

The premise that synergistic combinations might be usefully applied to overcome existing drug resistance was further explored using SPT in combination with the frontline anti-TB agents RIF and INH. In a two-dimensional (2D) pairwise screening assay, we evaluated two-drug permutations of RIF, INH, and SPT. The RIF-INH combination showed no interaction (see Fig. S5a in the supplemental material), while sub-MICs of INH (0.125 mg/liter) and RIF (0.003 mg/liter) reproducibly resulted in a modest (2-fold) reduction in the effective SPT concentration from 125 mg/liter to 62 mg/liter (Fig. S5b and c). To leverage the potential effect of SPT, a three-dimensional (3D) combination assay was performed in which RIF and INH were titrated against decreasing sub-MIC_90_ concentrations of SPT (1/2×, 1/4×, 1/8×, 1/16×, and 1/32× MIC_90_) using the format illustrated in Fig. S1b in the supplemental material. When tested against drug-susceptible M. tuberculosis H37Rv, RIF at sub-MIC ([RIF] = 0.003 mg/liter) plus SPT at both 1/4× and 1/2× MIC resulted in an 8-fold decrease in the effective concentration of INH from 0.25 mg/liter to 0.03 mg/liter (see Fig. S6 in the supplemental material). A kill kinetics assay showed that the addition of SPT to the RIF-INH combination elicited ∼1 log_10_ unit reduction (*P* < 0.001) in the viable bacillary population following 8-day exposure to the three-drug combination (see Fig. S7a in the supplemental material). In contrast, when the ΔRv1258c “tap” knockout mutant was tested, RIF at sub-MIC ([RIF] = 0.003 mg/liter) plus 1/2× MIC SPT resulted in only an ∼3-fold decrease in the effective concentration of INH, from 0.25 mg/liter to 0.09 mg/liter (Fig. S7b), again implicating Rv1258c in intrinsic antibiotic resistance in M. tuberculosis.

### The RIF-INH-SPT combination is active in M. tuberculosis-infected macrophages and against monoresistant pre-MDR strains.

Since M. tuberculosis survives and replicates in macrophages (42), the synergy of RIF-INH plus SPT was evaluated against intracellular bacilli in M. tuberculosis-infected THP-1 cells. This three-drug combination showed inhibitory activity at 1× MIC_90_ of the combined drugs (see Fig. S8 in the supplemental material). In contrast, the inhibitory effect was reduced when similar concentrations of each drug were applied individually or when the standard RIF-INH combination was used without SPT. The intracellular activity of this triple combination was further confirmed by CFU enumeration (see Fig. S9 in the supplemental material), which revealed a 2-log_10_ decrease in CFU/ml when 1× MIC_90_ RIF-INH-SPT was applied compared to the untreated control.

**FIG 3:**
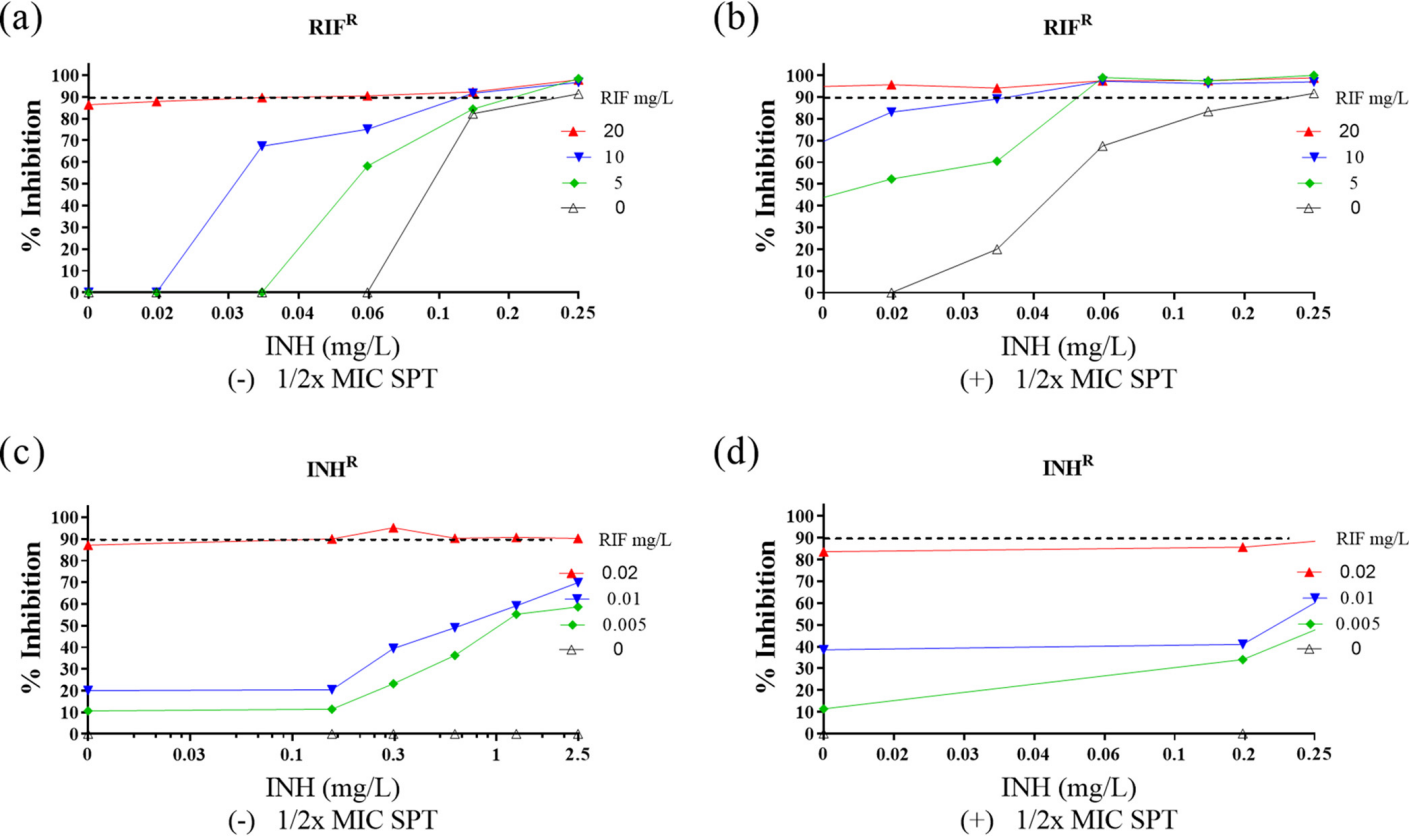
Activity against pre-MDR M. tuberculosis strains. *In vitro* activity of RIF-INH against RIF^r^ M. tuberculosis *rpoB*^S531L^ mutant in the absence (a) or presence (b) of 1/2× MIC SPT and against the INH^r^ M. tuberculosis *inhA* mutant in the absence (c) or presence (d) of 1/2× MIC SPT. The dashed horizontal line indicates 90% inhibition, and data are the means and standard deviations of two independent biological replicates.

To evaluate the efficacy of RIF-INH-SPT against known drug-resistant strains, the combination was tested against two pre-MDR M. tuberculosis mutants as follows: an RIF-monoresistant mutant carrying the common *rpoB* S531L allele and an INH-monoresistant strain harboring a −c15t mutation in the *inhA* promoter region that confers low-level INH resistance (Fig. 3). Duplicate checkerboard experiments showed that, for the *rpoB*^S531L^ (the S-to-L change at position 531 of RpoB) mutant, addition of 1/2× MIC SPT to the RIF-INH plate resulted in a decrease in the effective concentrations of both RIF (20 to 10 mg/liter) and INH (0.25 to 0.03 mg/liter) (Fig. 3a and b), indicating partial restoration of drug susceptibility in the presence of SPT. Enhanced susceptibility was also observed for the INH^r^ mutant, albeit to a lesser extent: a sub-MIC RIF concentration of 0.01 mg/liter and INH at 2.5 mg/liter achieved ∼80% bacterial inhibition (Fig. 3c and d).

## DISCUSSION

Notwithstanding recent promising claims (43), the bacterial capacity for acquisition of resistance by multiple mechanisms means that it is difficult, perhaps even conceivably impossible, to overcome antibiotic resistance sustainably (44). Different approaches can be used to circumvent resistance transiently, ensuring antibiotic efficacy despite the preexistence of resistant organisms in an infecting population. Combination therapy represents one such approach.

In a previous study, Chen et al. demonstrated a synergistic interaction between SQ109 and RIF when tested against RIF^r^ isolates: at 0.5× MIC, SQ109 was able to increase RIF’s activity against *de facto* resistant organisms in a dose-dependent manner (45). Recently, Yang et al. reported the enhanced efficacy of the imipenem-colistin combination against multiple drug-resistant Enterobacter cloacae bacteria *in vitro* and in an infection model (46). Relatively few studies have been undertaken to illustrate the association between potentiating drug interactions and the ability of the particular drug combination to overcome preexisting genetic resistance. In a clinical study, Ankomah et al. suggested that drugs acting synergistically can prevent treatment failure even when bacteria resistant to one of these drugs are present at the beginning of therapy (47). Our interaction studies between SPT and CPZ reaffirmed the susceptibility of SPT to Rv1258c-mediated efflux, an observation which suggests that efforts to modify SPT—including through novel chemical modifications that engineer resistance to efflux (18)—should be pursued.

We tested the susceptibility of the ΔRv1258c mutant to a small panel of antimycobacterial compounds with different mechanisms of action and observed that SPT alone was associated with hypersusceptibility. A similar hypersusceptibility phenotype was achieved against wild-type M. tuberculosis H37Rv via chemical potentiation of SPT using CPZ as the combination agent. However, the potentiating effect of CPZ was not specific to SPT and was instead observed for a handful of other agents. This suggests the likelihood that CPZ might disable more than one intrinsic resistance mechanism in M. tuberculosis. Further work is required to ascertain the precise mechanism for each compound, with evidence to date implicating multiple potential efflux systems in intrinsic resistance to INH, RIF, and the fluoroquinolones (48). For BDQ, the multisubstrate RND family transporter MmpS5-MmpL5 appears to be a strong candidate based on previous reports (49).

Our results revealed synergy between FA and SPT against drug-susceptible bacteria via a mechanism independent of the efflux inhibition seen with SPT-CPZ. Notably, the same FA-SPT combination exerted an enhanced inhibitory effect against a genotypically confirmed SPT^r^ mutant compared to an FA^r^ mutant. Although there is no definitive explanation for this finding, we postulate that the relative potency of FA (∼0.63 mg/liter) against the drug-susceptible H37Rv parent compared to that of SPT (∼50 mg/liter) could impact the FA-SPT combination against the SPT^r^ mutant, restoring susceptibility. A similar effect was not evident in an SPT-FA combination against the FA^r^ mutant, owing to the diminished activity of FA and high MIC_90_ of SPT. Of interest is the impact of individual active drugs in driving synergy.

Other explanations for potentiation include the sustained drug pressure emanating from the drug interactions, which leads to an increased effective dose of the drug combination. Moreover, some studies have demonstrated that the drug susceptibility of pathogens can be significantly enhanced as a result of a reduced efflux pump efficiency, either by genetic manipulation (50) or addition of efflux pump inhibitors (51, 52). The clinical relevance of this finding is that, despite the existence of bacterial resistance against a combination partner, it would still be possible to achieve optimal therapeutic outcomes via the use of appropriate potent drug combinations.

Previous work has demonstrated the potential of having three-drug combinations when compared to individual or two-drug regimens (53, 54). Recently, Tekin et al. reported that combinations of three different antibiotics can often overcome antimicrobial resistance to antibiotics, even when none of the three antibiotics on their own—or even two of the three together—is effective (55). In addition, based on drug interaction studies, Ramon-Garcia et al. hypothesized that the synergistic activity of the triplet combination might have multiplicative effects (10). Here, SPT was deployed as part of a three-drug regimen, which also included RIF and INH, the two drugs that form the cornerstone of TB treatment. Other studies have shown the interaction between RIF and INH against M. tuberculosis to have no interaction or to be mildly antagonistic (8, 56). The inclusion of SPT in this drug regimen was underpinned by reports that 24 out of 70 random combinations tested were synergistically active in M. smegmatis (10). This suggests a large unexplored pool of synergistic combinations. Notably, SPT exhibited synergy with several compounds both *in vitro* and *ex vivo* (10), even though the compound has a high MIC_90_ against M. tuberculosis when administered on its own (18).

In the three-drug combination assay, synergy resulted when subinhibitory concentrations (1/2× and 1/4× MICs) of SPT were titrated into media containing RIF and INH. This finding correlated well with the results of time-kill kinetics. However, the time-kill assay suggested that the inhibitory effect of the three-drug interaction was bacteriostatic (≤3 log_10_ CFU reduction) and not bactericidal. This observation reveals that the combination of RIF and INH—two bactericidal drugs that are most potent against actively dividing cells—shows bacteriostatic effects. Furthermore, the inhibition of growth induced by a bacteriostatic drug, SPT, results in an overall static effect when the drug is used in combination with a bactericidal drug. Other studies have shown that, in similar interactions, the resulting effect achieves a more efficient clearance at lower concentrations (31).

In attempting to exploit synergy for potential optimal treatment outcomes, an investigation of the RIF-INH plus SPT interaction was performed in *rpoB* and *inhA* mutants. The RIF^r^ *rpoB* mutant had an MIC value of >2,000 times the MIC_90_ for drug-susceptible M. tuberculosis. Notably, addition of 1/2× MIC of SPT restored partial drug efficacy against this resistant mutant. As with the drug-susceptible H37Rv strain, a mechanistic explanation for the synergy observed using the RIF-INH plus SPT combination against the *rpoB* mutant is presently lacking. INH targets mycobacterial cell envelope biosynthesis, possibly enhancing permeation of SPT into the bacilli. However, access alone may not necessarily contribute to the synergistic interaction. Chen et al. reported synergy between SQ109, a presumed cell envelope inhibitor, and RIF (45). Conversely, EMB, which also affects mycobacterial cell envelope synthesis, did not exhibit synergy with RIF (57).

Prior reports have shown RIF to be an efficient inducer of cytochrome P450 (CYP 450), a superfamily of heme-containing enzymes involved in the biosynthesis of compounds, such as sterols, steroids, and fatty acids, as well as detoxification of xenobiotics and chemicals (58). RIF has been linked with the induction of CYP both in humans and in M. tuberculosis (59). The elevated levels of CYP have been associated with drug resistance due to the enhanced rate of elimination of the drugs by metabolism and detoxification pathways. INH, conversely, inhibits CYP in M. tuberculosis (59). This ability of INH to inhibit CYP may contribute to synergy in the RIF-INH plus SPT combination when the active form of INH is not rapidly eliminated inside M. tuberculosis and when SPT acts by further reducing the activity of CYPs.

There are prospects to combine SPT with RIF-INH in treatment regimens. SPT is given by intramuscular injection to achieve therapeutic concentrations in serum of about 100 mg/liter 1 h after a single 2-g dose. An over 4-fold increase in its effectiveness within a triple SPT-RIF-INH combination, as indicated by these data, would potentially allow for oral formulation, a critical delivery format when administering treatment to TB outpatients. In summary, these *in vitro* and *ex vivo* results suggest that the RIF-INH plus SPT triple-combination may be an effective therapeutic option for the treatment of both drug-susceptible and -resistant M. tuberculosis infections. They also reinforce a growing body of evidence supporting the utility of drug potentiation strategies in improving treatment outcomes.

## MATERIALS AND METHODS

### Chemicals and reagents.

All chemicals and solvents were purchased from Sigma-Aldrich. Working solutions of all antimicrobial agents were prepared in dimethyl sulfoxide (DMSO).

### Bacterial strains and culture conditions.

The laboratory strain, M. tuberculosis H37Rv, its derivative mutants, and a reporter strain that has been used previously in high-throughput antimicrobial drug screening and constitutively expresses green fluorescent protein (GFP), H37Rv pMSP::eGFP (41), were maintained as freezer stocks. Strains were inoculated in standard Middlebrook 7H9 medium supplemented with 10% oleic acid-albumin-dextrose-catalase (OADC) (Difco) and incubated as stationary cultures at 37°C for approximately 3 days, subcultured, and incubated until culture density was an OD_600_ of ∼0.5. A second reporter mutant, M. tuberculosis H37Rv::(pSMYC::mCherry) (60), which constitutively expresses the mCherry fluorophore, was grown in medium containing 50 mg/liter hygromycin. Cell suspensions were diluted to give an expected final concentration of 10^5^ cells/ml at the time of inoculation into the microplate for the MIC assays.

### Drug susceptibility assays.

The resazurin microtiter assay (REMA) was used to determine the susceptibility of drugs against M. tuberculosis strains as described (61). Briefly, 2-fold serial dilutions of compounds were performed on clear, round-bottom 96-well plates using 7H9-OADC medium. M. tuberculosis cultures, grown to an OD_600_ of 0.5 (∼10^8^ cells/ml) and diluted 1,000-fold, were added at equal volume for a total volume of 100 μl per well. The plates were sealed in zip-lock bags and incubated at 37°C for 7 days, consistent with EUCAST guidelines (62) and published literature (63) recommending that MIC plates should be read after 7 and 14 days postinoculation. Resazurin dye was added and the plates incubated for a further 24 h. Fluorescence readings, at excitation and emission wavelengths of 540 and 590 nm, respectively, were recorded using a BMG Labtech POLARstar Omega plate reader (BMG Labtech, Offenburg, Germany) or a SpectraMax i3x plate reader (Molecular Devices). The lowest drug concentration that inhibited growth by at least 90% was determined from the dose-response curve; this concentration was defined as the MIC_90_ value.

### Checkerboard assays. (i) 2D checkerboard.

Standard “two-dimensional” (2D) drug-drug interactions were determined by checkerboard titration in a 96-well plate (see Fig. S1a in the supplemental material). The 2D microdilution was carried out as described (45) with slight modification. Briefly, the first drug (A) to be serially diluted was dispensed (2 μl) along the *x* axis (columns 3 to 11; row B) at a starting concentration 100 times higher than the final concentration in the well, and 2 μl per well of the second drug (B) was serially dispensed along the *y* axis (from row B to H) at a starting concentration 100 times higher than the final concentration in the 96-well microtiter plate. The first column (column 1) and last column (column 12) contained drug-free controls (with 1% DMSO as a diluent) and a control drug concentration giving maximum inhibition, respectively. The second column from B2 to H2 and first row from A3 to A11 contained individual drugs, thus providing the MIC for each drug alone in each assay (each plate). The plates were placed in zip-lock bags and incubated at 37°C for 7 days. Resazurin dye was then added and the plates incubated for a further 24 h. A visible color change from blue to pink indicated growth of bacteria, and the visualized MIC was defined as the lowest concentration of drug that prevented growth (at which the color change did not occur) (61). Fluorescence readings (excitation, 544 nm; emission, 590 nm) were obtained using a BMG Labtech POLARstar Omega plate reader (BMG Labtech, Offenburg, Germany) or a SpectraMax i3x plate reader (Molecular Devices). The mean fluorescence value for the “maximum inhibition” wells (column 12) was subtracted from all other wells to control for background fluorescence. Percent inhibition was defined as 1 − (test well fluorescence units/mean fluorescence units of maximum inhibition wells) × 100 on day 8 after incubation. The lowest drug concentration effecting inhibition of 90% was considered the MIC_90._ In addition, synergy was interpreted according to the sum of fractional inhibitory concentration (ΣFIC). The fractional inhibitory concentration for each compound was calculated as follows (64): FIC_A_ − (MIC of compound A in the presence of compound B)/(MIC of compound A alone), where FIC_A_ is the fractional inhibitory concentration of compound A. Similarly, the FIC for compound B was calculated. The ΣFIC was calculated as FIC_A_ + FIC_B_. Synergy was defined by values of ΣFIC of ≤0.5, antagonism by ΣFIC of >4.0, and no interaction by ΣFIC values from 0.5 to 4.0 (30).

### (ii) 3D checkerboard.

In the three-drug (“three-dimensional” [3D]) combinations (Fig. S1b), microdilutions for the first two drugs were initially set up principally following the standard 2D checkerboard assay protocol described above. The third drug (2 μl) was then added at a starting concentration 100 times higher than the final concentration in the well as an overlay at five subinhibitory concentrations ranging from 1/32 to 1/2 of the single-drug MIC. Well A2 on all plates contained the third drug only, providing the single-drug MIC for the third drug in each assay (set of 5 plates). After inoculation with a log-phase culture, an OD_600_ of 0.5 (∼10^8^ cells/ml) of M. tuberculosis, 50 μl to each well, the plates were placed in zip-lock bags and incubated for 7 days at 37°C before addition of resazurin. The plates were further incubated for 24 h, and the results were read in the BMG Labtech plate reader (excitation, 544 nm; emission, 590 nm) (63). The percent inhibition was calculated as described above.

### Macrophage assays. (i) Cell culture and maintenance.

Human promonocytic THP-1 cells were maintained in RPMI 1640 medium (Sigma) supplemented with 10% fetal bovine serum (FBS) (Invitrogen) at an initial density of 8 × 10^5^ cells/ml at 37°C in a humidified, 5% CO_2_ atmosphere. Prior to plating of the cells, viability was assessed by trypan blue exclusion method (65). Maturation of THP-1 cells into macrophages was induced by adding 200 nM PMA (phorbol 12-myristate 13-acetate; Sigma) in cell culture medium for 24 h. Differentiated macrophages were then washed three times with prewarmed phosphate-buffered saline (PBS) to remove the PMA and replenished with cell culture medium.

### (ii) Infection of macrophages and drug treatment.

To check the efficacy of drugs in macrophages, 5 × 10^4^ THP-1 cells/well (100-μl final volume) in 96-well flat-bottomed tissue culture plates were differentiated into macrophages. To infect macrophages, an exponentially growing M. tuberculosis H37Rv::(pSMYC::mCherry) culture was harvested by centrifugation and washed twice with PBS. The pellet was resuspended in PBS and passed through a 5-μm filter to generate a suspension of single-cell bacilli. The bacterial suspension density was estimated by measuring OD at 600 nm, corresponding an OD_600_ of ∼0.5 to 1 × 10^8^ CFU/ml. Infection medium comprised cell culture medium containing a number of bacteria required to achieve a multiplicity of infection (MOI) of 5:1 (5 bacilli for every THP-1 cell) (66). The macrophage cells were overlaid with infection medium and incubated at 37°C in 5% CO_2_ for the phagocytic period of 3 h. Cells were then washed gently and thoroughly with prewarmed PBS to remove extracellular bacteria. The cells from three wells were lysed by adding Triton X-100 (0.05% in PBS), and the lysate was plated onto 7H10 to score CFU for untreated day zero. The cells in remaining wells were treated with the indicated antibiotic either alone or in combination at 1× MIC_90_ or 5× MIC_90_ as determined in liquid culture. Hygromycin at 50 mg/liter was added into the cell culture medium for all wells to maintain the plasmid expressing mCherry.

### (iii) Fluorescence measurement.

The fluorescence of the mCherry reporter (excitation, 590 nm; emission, 610 nm) was measured at different time points on a BMG Labtech plate reader.

### (iv) CFU enumeration.

To estimate the numbers of live bacilli after drug treatment, untreated and drug-treated M. tuberculosis-infected cells were lysed in Triton X-100 (0.05% in PBS) on days 2, 4, and 6, and serial dilutions of the cell lysate were plated onto 7H10 agar. Colonies were counted after 3 to 4 weeks of incubation at 37°C.

### Statistical analyses.

Statistical analyses were performed using Prism 9.0.0.121 (GraphPad). Means were compared via analysis of variance (ANOVA) with posttest evaluation using Dunnett's or Bonferroni's test. *P* values are abbreviated as follows: *, *P *< 0.05; **, *P *< 0.01; ***, *P *< 0.001.

## Supplementary Material

Supplemental file 1
