# Supplementary material for: Developing Synergistic Drug Combinations To Restore Antibiotic Sensitivity in Drug-Resistant Mycobacterium tuberculosis
Source: Antimicrob Agents Chemother. 2021 Apr 19;65(5):e02554-20. doi: 10.1128/AAC.02554-20 (PMC8092878; doi:10.1128/AAC.02554-20)
Supplement: Supplemental file 1 [file AAC.02554-20-s0001.pdf]

SUPPLEMENTARY INFORMATION

Developing synergistic drug combinations to restore antibiotic sensitivity in drug-resistant *Mycobacterium tuberculosis*

Charles Omollo,[a,b,c,d,#] Vinayak Singh,[a,b,c,d] Elizabeth Kigondu,[a,b,c,*] Antonina Wasuna,[a,b,c] Pooja Agarwal,[c,d] Atica Moosa,[c,d] Thomas R. Ioerger,[e] Valerie Mizrahi,[c,d,f] Kelly Chibale,[a,b,d] and Digby F. Warner[c,d,f, #]

a. Department of Chemistry, University of Cape Town, Rondebosch 7701, South Africa;
b. South African Medical Research Council Drug Discovery and Development Research Unit, University of Cape Town, Rondebosch 7701, South Africa;
c. SAMRC/NHLS/UCT Molecular Mycobacteriology Research Unit, DST/NRF Centre of Excellence for Biomedical Tuberculosis Research, Department of Pathology, University of Cape Town, Rondebosch 7701, South Africa;
d. Institute of Infectious Disease & Molecular Medicine, University of Cape Town, Rondebosch 7701, South Africa;
e. Texas A&M University, Department of Computer Science, College Station, TX, 77843, USA
f. Wellcome Centre for Infectious Diseases Research in Africa, University of Cape Town, Rondebosch 7701, South Africa.

*Current address: *Centre for Traditional Medicine & Drug Research, Kenya Medical Research Institute, Nairobi, Kenya*

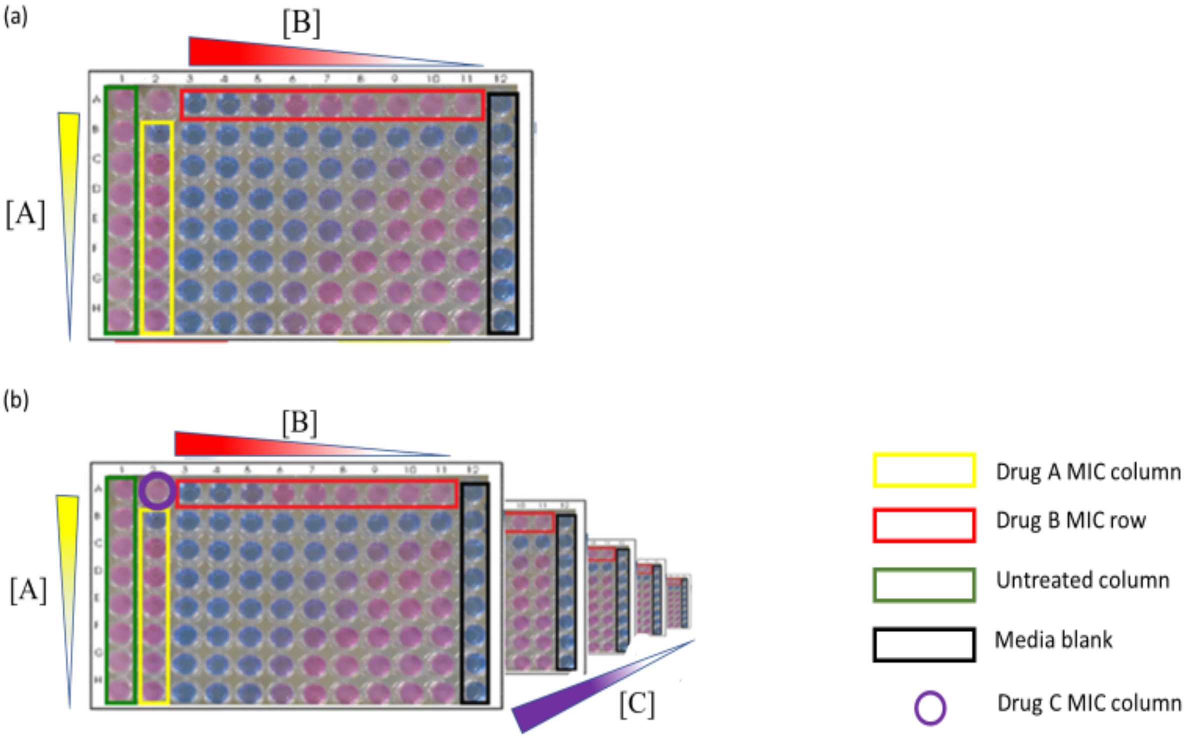

Figure S1. Schematic representation of (a) 2D and (b) 3D checkerboard 96-well plate assays. In each assay, the MICs of individual drugs are determined in the “Drug MIC” row/column. The lowest sum FIC (ΣFIC) is calculated from wells containing the combined drugs at different concentrations. For the 3D assay, a third drug [C], is added as an overlay at sub-MIC (five concentrations from 1/2× to 1/32×) on each of the five 96-well plates in the stack.

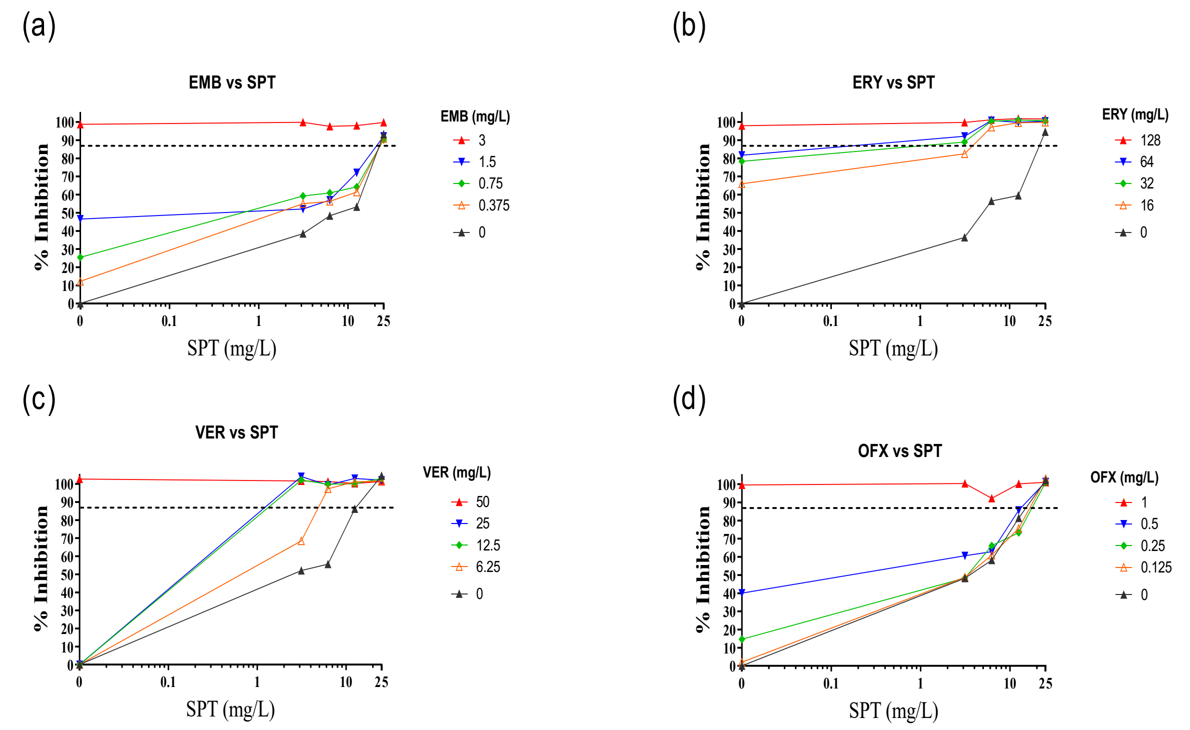

(a)
EMB vs SPT
EMB (mg/L)
3
1.5
0.75
0.375
0
% Inhibition
SPT (mg/L)
(b)
ERY vs SPT
ERY (mg/L)
128
64
32
16
0
% Inhibition
SPT (mg/L)
(c)
VER vs SPT
VER (mg/L)
50
25
12.5
6.25
0
% Inhibition
SPT (mg/L)
(d)
OFX vs SPT
OFX (mg/L)
1
0.5
0.25
0.125
0
% Inhibition
SPT (mg/L)

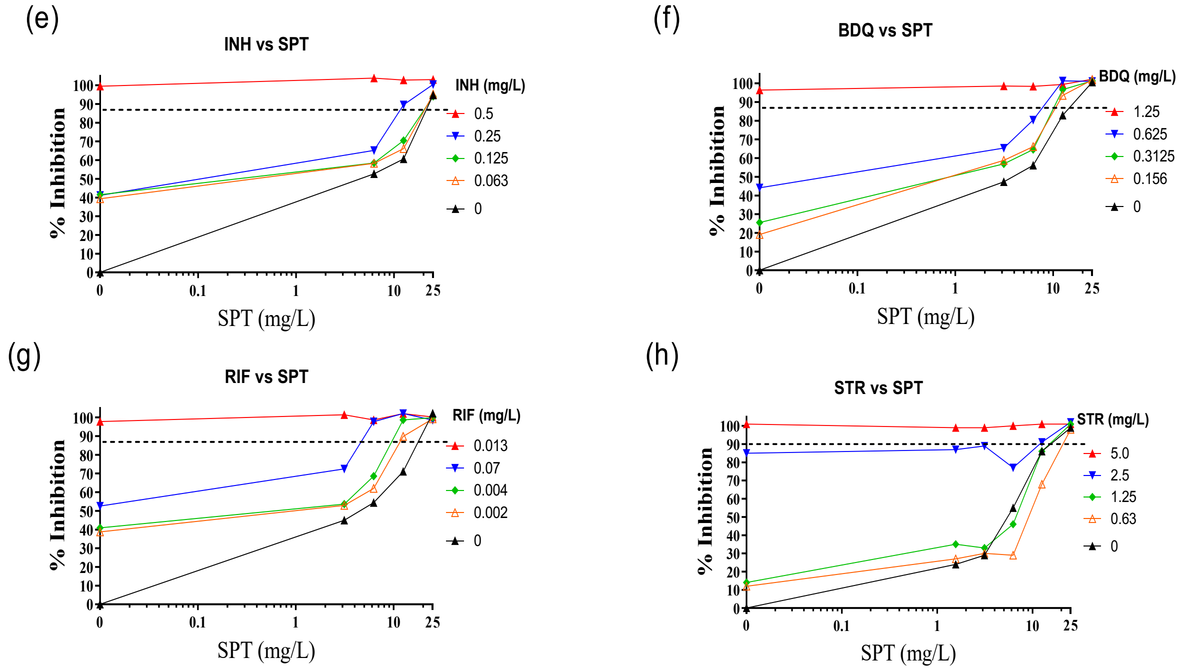

(e)
INH vs SPT
INH (mg/L)
0.5
0.25
0.125
0.063
0
% Inhibition
SPT (mg/L)
(f)
BDQ vs SPT
BDQ (mg/L)
1.25
0.625
0.3125
0.156
0
% Inhibition
SPT (mg/L)
(g)
RIF vs SPT
RIF (mg/L)
0.013
0.07
0.004
0.002
0
% Inhibition
SPT (mg/L)
(h)
STR vs SPT
STR (mg/L)
5.0
2.5
1.25
0.63
0
% Inhibition
SPT (mg/L)

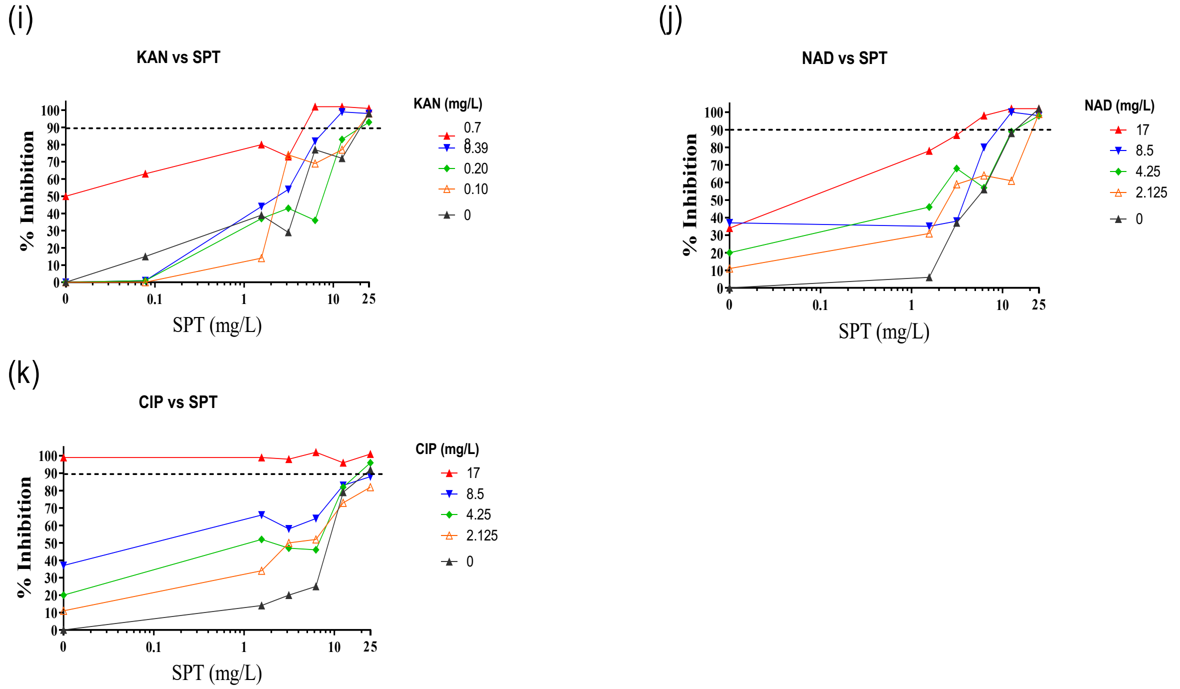

Figure S2. *In vitro* interactions between SPT and selected compounds against wild-type *M. tuberculosis* H37Rv. Dashed horizontal lines indicate 90% inhibition. Data are the means and standard deviations of two independent biological replicates. BDQ, bedaquiline; CIP, ciprofloxacin; EMB, ethambutol; ERY, erythromycin; INH, isoniazid; KAN, kanamycin; NAD, nalidixic acid; OFX, ofloxacin; RIF, rifampicin; SPT, spectinomycin; STR, streptomycin; VER, verapamil.

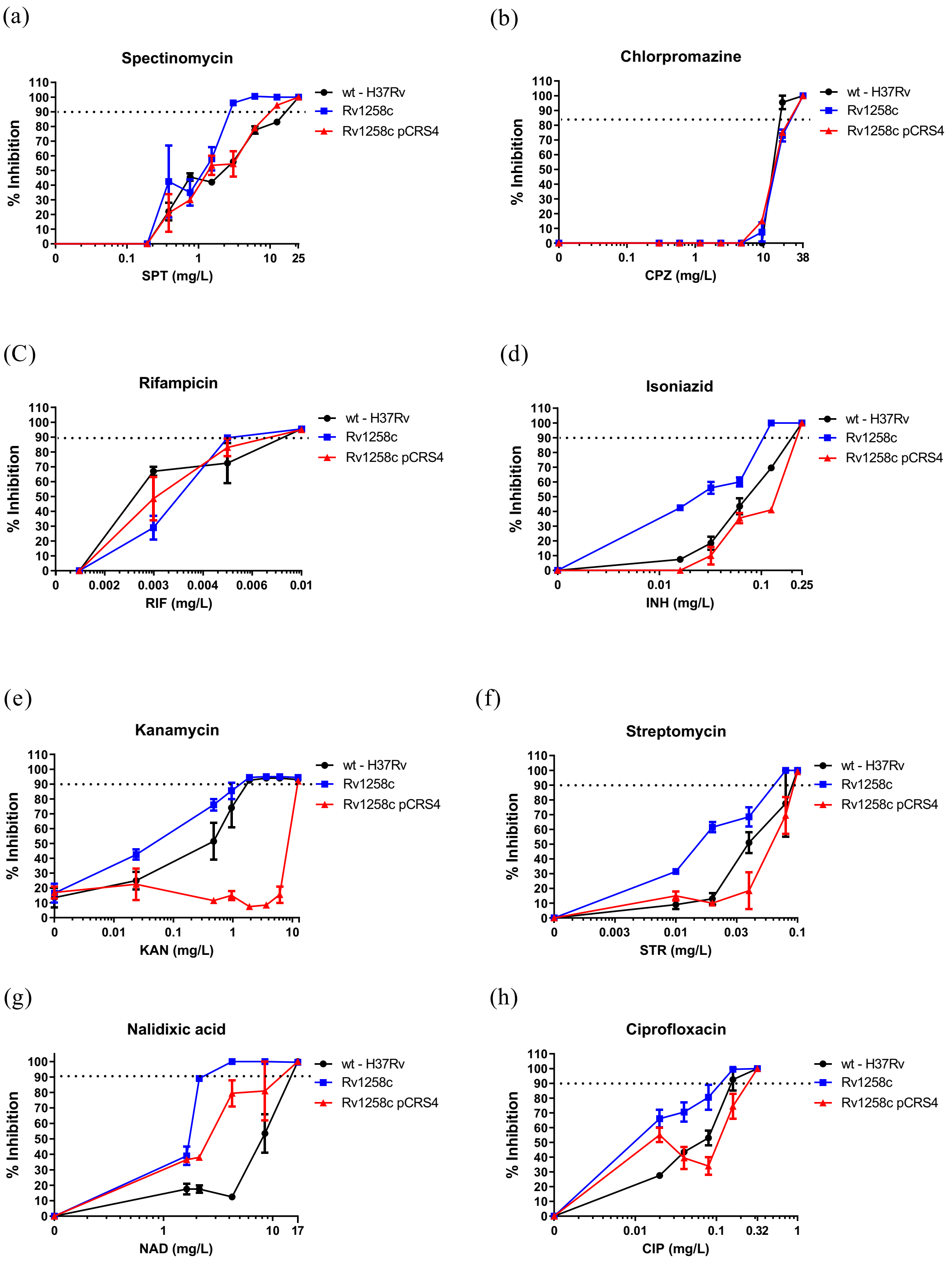
(a)
Spectinomycin
% Inhibition
SPT (mg/L)
wt - H37Rv
Rv1258c
Rv1258c pCRS4
(b)
Chlorpromazine
CPZ (mg/L)
(C)
Rifampicin
RIF (mg/L)
(d)
Isoniazid
INH (mg/L)
(e)
Kanamycin
KAN (mg/L)
(f)
Streptomycin
STR (mg/L)
(g)
Nalidixic acid
NAD (mg/L)
(h)
Ciprofloxacin
CIP (mg/L)

(i)

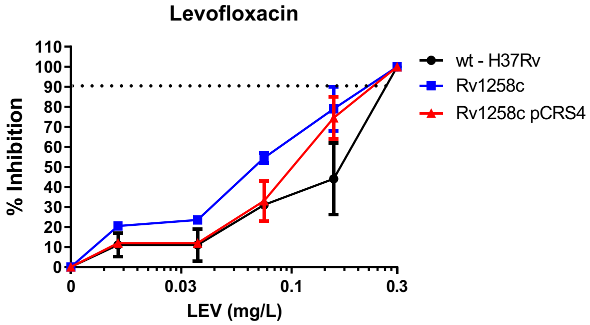

Figure S3. Investigating potential hypersensitivity of the *M. tuberculosis* Δ*Rv1258c* mutant to different anti-TB compounds. All compounds were tested against wild-type H37Rv, the "tap" knock-out (Δ*Rv1258c*), and its complemented derivative (Δ*Rv1258c* pCRS4) in 96-well plates. Bacterial viability was assessed by fluorescence-based resazurin microtiter assay. Dashed horizontal lines indicate 90% inhibition. Data are the means and standard deviations of two independent biological replicates. CIP, ciprofloxacin; INH, isoniazid; KAN, kanamycin; NAD, nalidixic acid; OFX, ofloxacin; RIF, rifampicin; SPT, spectinomycin; STR, streptomycin; CPZ, chlorpromazine; LEV, levofloxacin.

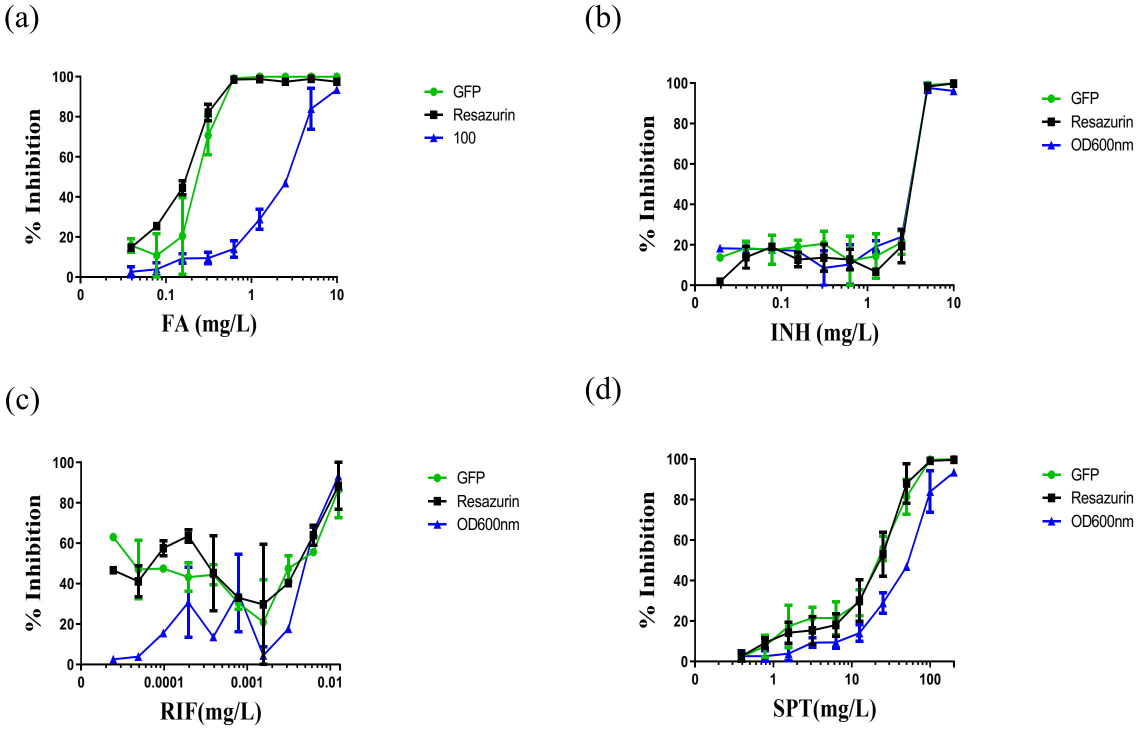

Figure S4. Comparison of GFP, resazurin and absorbance (OD_{600}) assays in calculating MIC values against wild-type *M. tuberculosis* H37Rv. A 96-well based assay was performed with selected panel of drugs (a) FA, fusidic acid; (b) INH, isoniazid; (c) RIF, rifampicin; (d) SPT, spectinomycin. After 8-days of incubation, fluorescence intensities of both GFP and resazurin assays were determined alongside OD_{600nm} readings. These were calculated and presented as % inhibitions. Data are the means and standard deviations of two independent biological replicates.

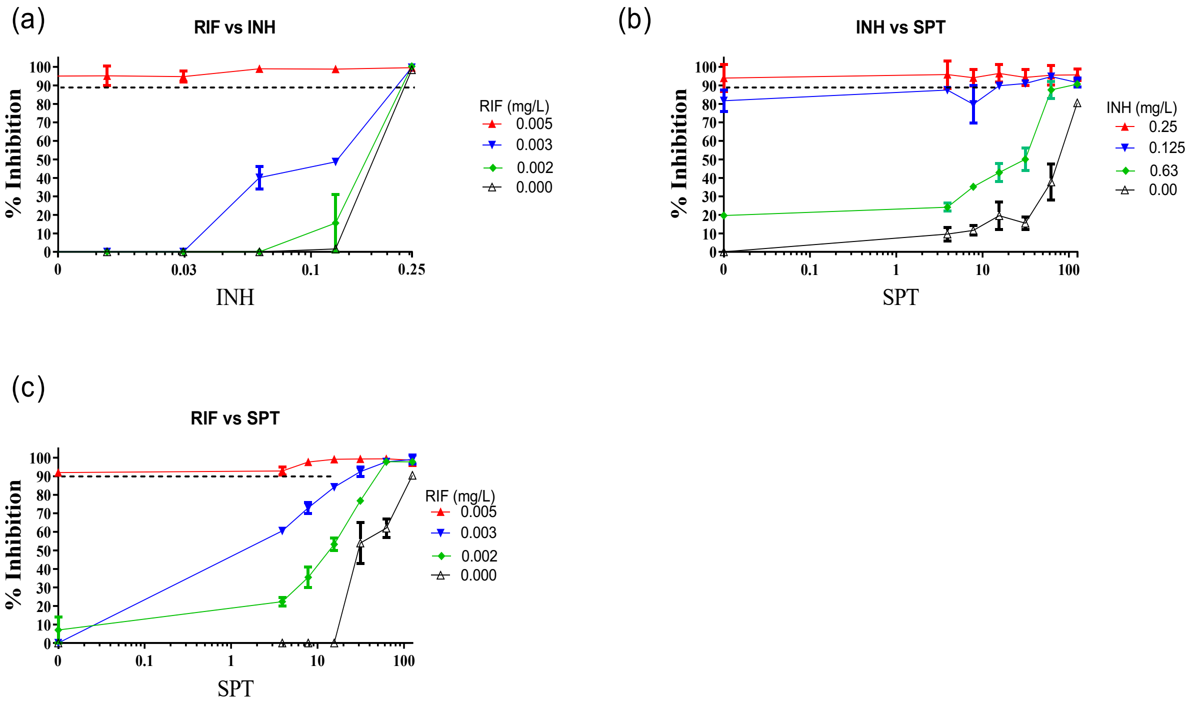

Figure S5. *In vitro* interactions between (a) RIF and INH, (b) INH and SPT, and (c) RIF and SPT in 2D checkerboard assays against wild-type *M. tuberculosis* H37Rv. Bacterial viability was assessed by fluorescence-based resazurin microtiter assay. Dashed horizontal lines indicate 90% inhibition. Data are the means and standard deviations of two independent biological replicates.

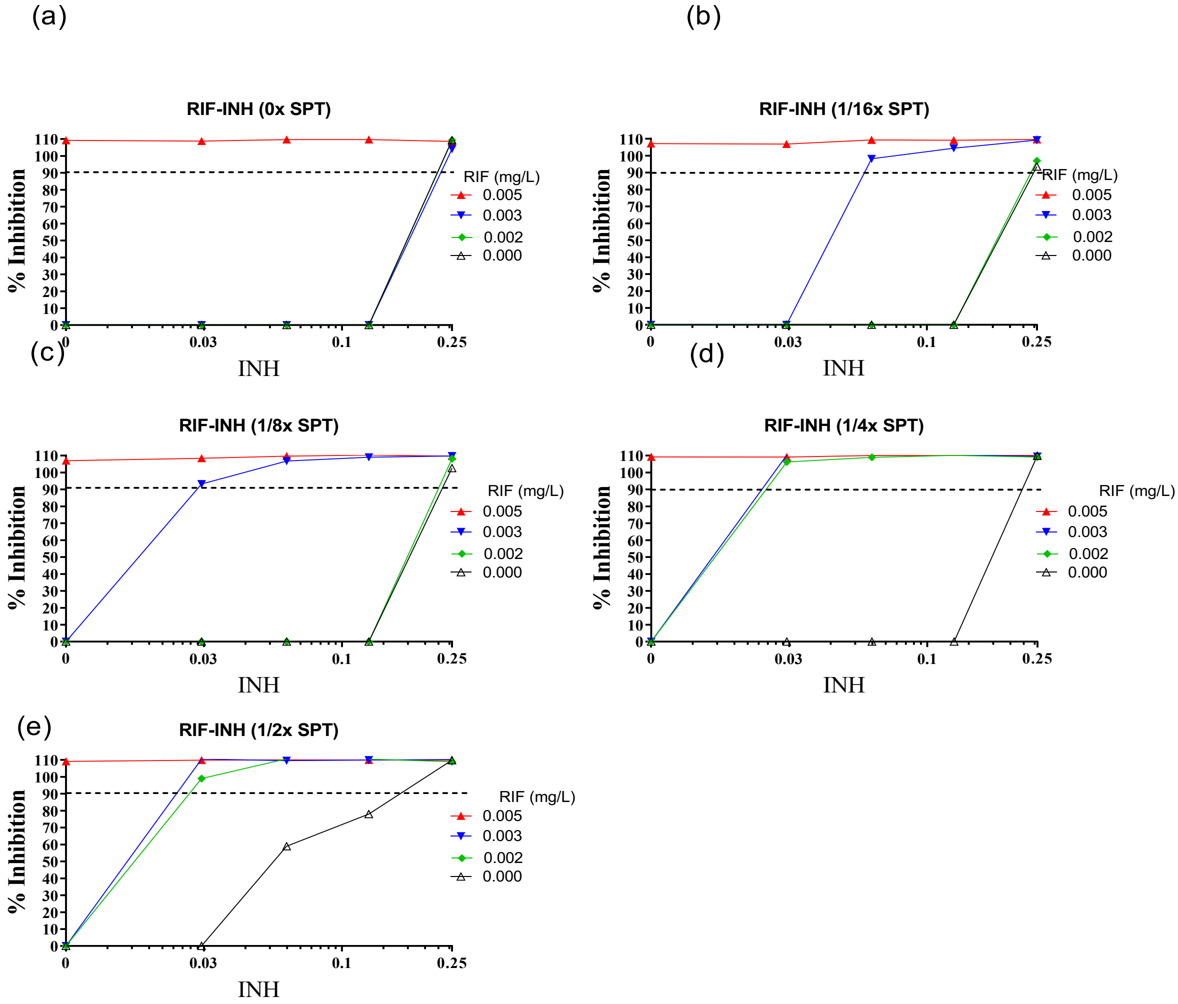

Figure S6. 3D checkerboard assay of RIF and INH against wild-type *M. tuberculosis* H37Rv in the presence of sub-MIC_{90} concentrations of SPT: (a) 0x SPT, (b) 1/16× SPT, (c) 1/8× SPT, (d) 1/4× SPT, and (e) 1/2× SPT. Bacterial viability was assessed in two independent experiments by fluorescence-based resazurin microtiter assay. Dashed horizontal lines indicate 90% inhibition.

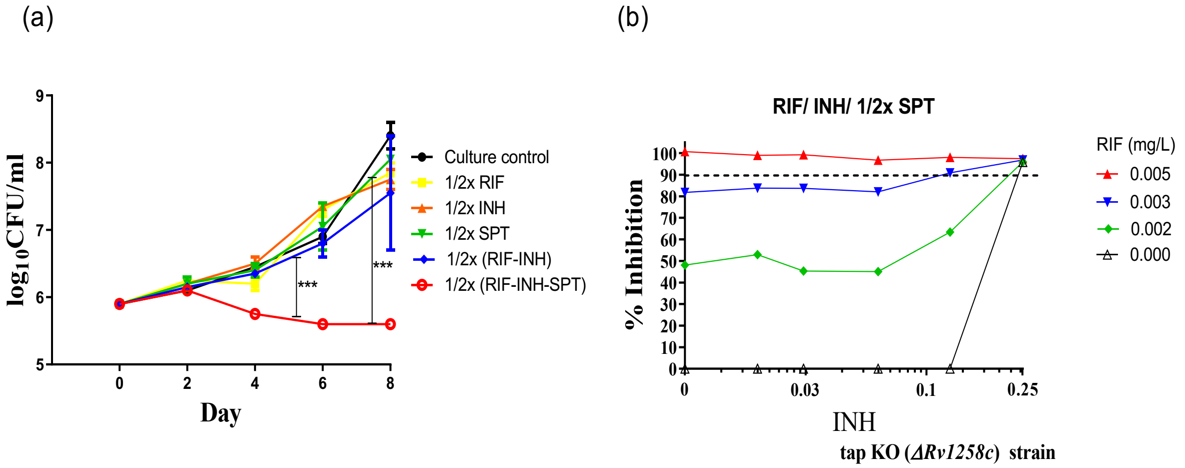

Figure S7. *In vitro* interactions between RIF and INH in the presence of a sub-inhibitory concentration of SPT (1/2× MIC SPT). (*a*) Kill kinetics determined following exposure of wild-type *M. tuberculosis* H37Rv to sub-inhibitory concentrations of RIF, INH and SPT individually and in combination. Viable cell counts were determined at the indicated timepoints by plating and enumerating colony forming units (CFU) on antibiotic-free Middlebrook 7H10 agar ($p < 0.001$). (*b*) Checkerboard assays were performed using RIF, INH and 1/2×MIC SPT against the Δ*Rv1258c* mutant ("tap KO strain") in a 96-well microtiter plate. The fluorescence reading was obtained on Day 8 following the addition of resazurin. Data are representative of two independent biological replicates.

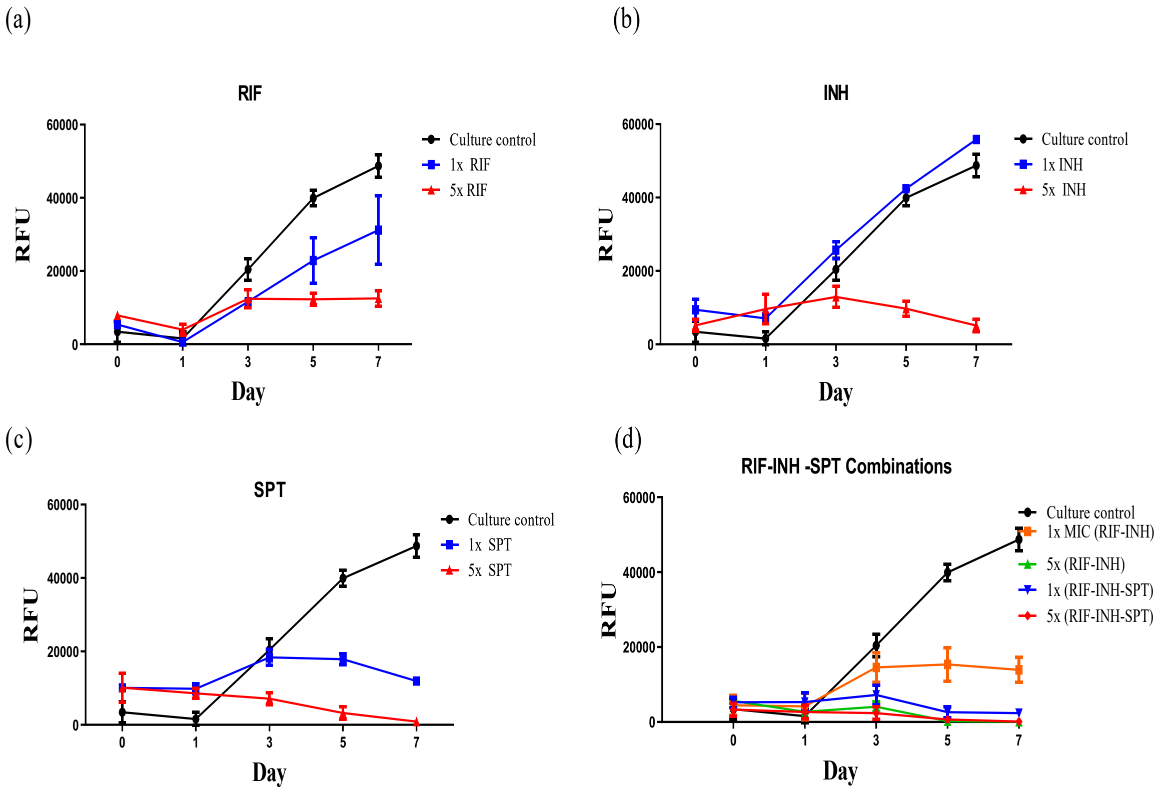

Figure S8. Activities of RIF, INH and SPT alone and in combination in a model of *M. tuberculosis* intracellular infection. THP-1 cells were infected with exponentially growing *M. tuberculosis* H37Rv pSMYC::mCherry) at a multiplicity of infection (MOI) of 1:5 cells:bacilli. Following infection, cells were exposed to different concentrations of each of the partner drugs alone or in combination. The plots detail the inhibitory activities of the drugs as determined by mCherry fluorescence intensity. Data are the means and standard deviations of independent biological experiments performed in triplicate.

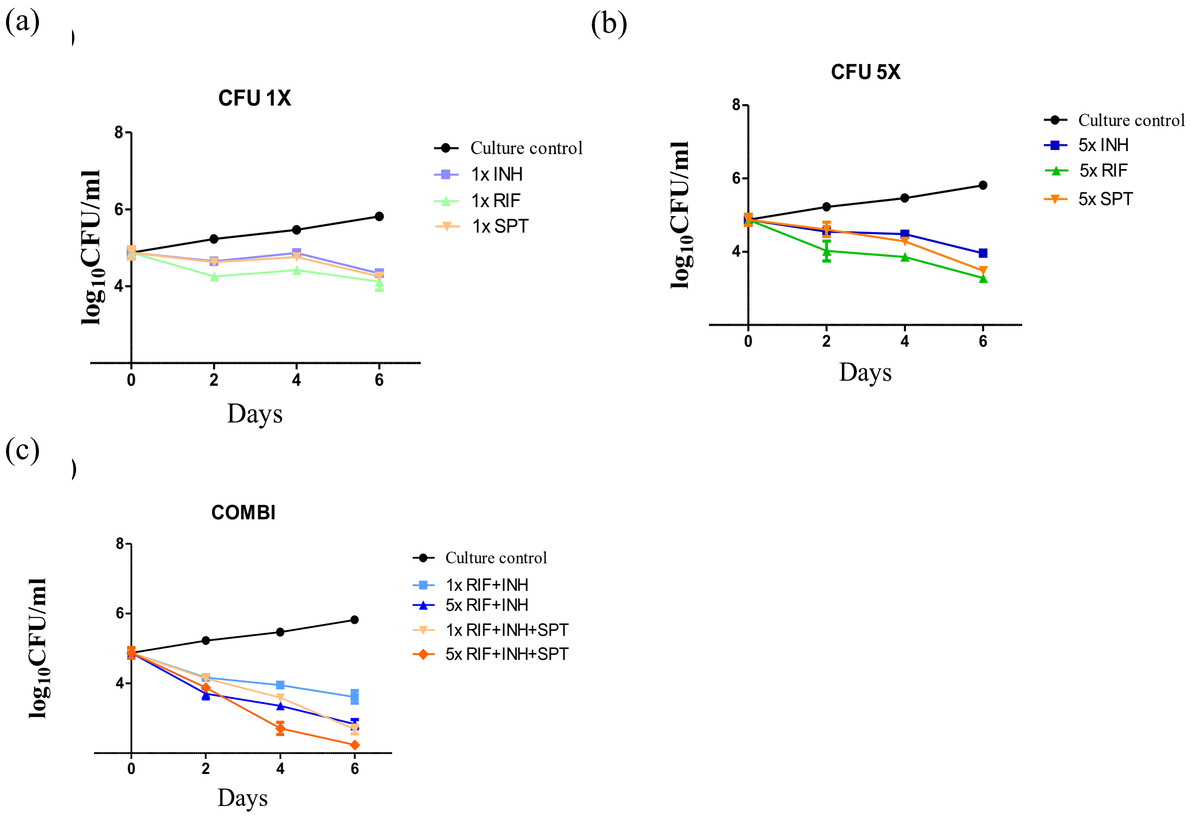

Figure S9. Intracellular activities of RIF, INH and SPT alone and in combination. THP-1 cells were infected with *M. tuberculosis* H37Rv at a multiplicity of infection (MOI) of 1:5 cells:bacilli. Following infection, cells were exposed to different concentrations of each of the partner drugs alone (“CFU 1X”, “CFU 5X”) or in combination (“COMBI”). The plots detail the inhibitory activities of the drugs as determined by CFU enumeration following plating on antibiotic-free Middlebrook 7H10 solid medium. Data are the means and standard deviations of independent biological experiments performed in triplicate.

Table S1 *In vitro* activities of selected compounds against wild-type *M. tuberculosis* H37Rv

			MIC$_{90}$ (mg/L)*				
**Compound**	**H37Rv**	**FA^R**	**SPT^R**	***ΔRv1258c*†**	***ΔRv1258c* pCRS4**	***inhA***	***rpoB***
FA	0.63	>248	0.63	0.32	0.63		
SPT	50-100	125	>8000	**3.9**	31		50
‘1599	3.12			3.12-1.56	6.25		
CPZ	22.2	22.2	22.2	22.2	22.2		
RIF	0.01		0.01	0.005	0.01	0.01	20
INH	0.25		0.125	0.125	0.25	>4.0	0.06
KAN	1.56			1.56	12.5		
LEV	0.31			0.16-0.31	0.31		
NAD	17			8.5-17	17-34		
CIP	0.16			0.16	0.32		
STR	0.08			0.08	0.08-0.16		
BDQ	1.2			0.6	1.2		

*The MIC$_{90}$ was defined as the lowest drug concentration which inhibited growth by at least 90%.

†MIC$_{90}$ values in bold type indicate hypersensitivity of the Δ*Rv1258c* mutant to the specific compound(s)

Table S2: MIC_{90} and ΣFIC values for selected antimycobacterial compounds used against wild-type *M. tuberculosis* H37Rv alone and in combination with CPZ

Compound	MIC_{90} (mg/L)*, alone	MIC_{90} (mg/L)*, in combination	FIC	ΣFIC†
RIF	0.0032	0.00037	0.116	**0.37**
CPZ	22	5.50	0.250	
INH	0.38	0.094	0.247	**0.50**
CPZ	22	5.50	0.250	
KAN	7.16	3.57	0.499	1.00
CPZ	22	11	0.500	
STR	1.15	0.14	0.122	0.62
CPZ	22	11	0.500	
BDQ	3.00	0.0059	0.002	**0.25**
CPZ	11	2.25	0.250	
NAD	14	1.8	0.125	**0.25**
CPZ	22	2.75	0.125	
CIP	2.50	1.27	0.508	0.76
CPZ	22	5.50	0.250	
LEV	2.30	2.30	1.00	2.00
CPZ	22	22	1.00	

*The MIC_{90} was defined as the lowest drug concentration which inhibited growth by at least 90%.

†Bold type indicates synergistic combinations (ΣFIC ≤ 0.5)

Table S3: MIC_{90} and ΣFIC values for selected antimycobacterial compounds used against wild-type *M. tuberculosis* H37Rv alone and in combination with SPT

**Drugs/Compound**	**MIC_{90} (mg/L)*, alone**	**MIC_{90} (mg/L)*, in combination**	**FIC**	**ΣFIC†**
RIF	0.013	0.002	0.015	0.52
SPT	25	12.5	0.500	
INH	0.5	0.25	0.500	1.00
SPT	25	12.5	0.500	
BDQ	1.25	0.156	0.125	0.63
SPT	25	12.5	0.500	
OFX	1.0	0.5	0.500	1.50
SPT	25	25	1.000	
EMB	3.00	1.5	0.500	1.50
SPT	25	25	1.000	
ERY	128	32	0.125	**0.38**
SPT	25	6.25	0.250	
VER	50	6.25	0.125	**0.25**
SPT	25	6.25	0.125	
KAN	1.56	1.56	1.000	1.25
SPT	25	6.125	0.250	
STR	0.08	0.16	2.000	2.50
SPT	25	12.5	0.500	
CIP	0.16	0.32	2.000	2.25
SPT	25	6.25	0.250	
NAD	17	34	2.000	2.13
SPT	25	6.25	0.125	

*The MIC_{90} was defined as the lowest drug concentration which inhibited growth by at least 90%.

†Bold type indicates synergistic combinations (ΣFIC ≤ 0.5)
